# A Versatile and Efficient Method to Isolate DNA–Polymer Conjugates

**DOI:** 10.1021/acsmacrolett.3c00371

**Published:** 2023-09-01

**Authors:** Nico Alleva, Katharina Eigen, David Y. W. Ng, Tanja Weil

**Affiliations:** Max Planck Institute for Polymer Research, Ackermannweg 10, 55128 Mainz, Germany

## Abstract

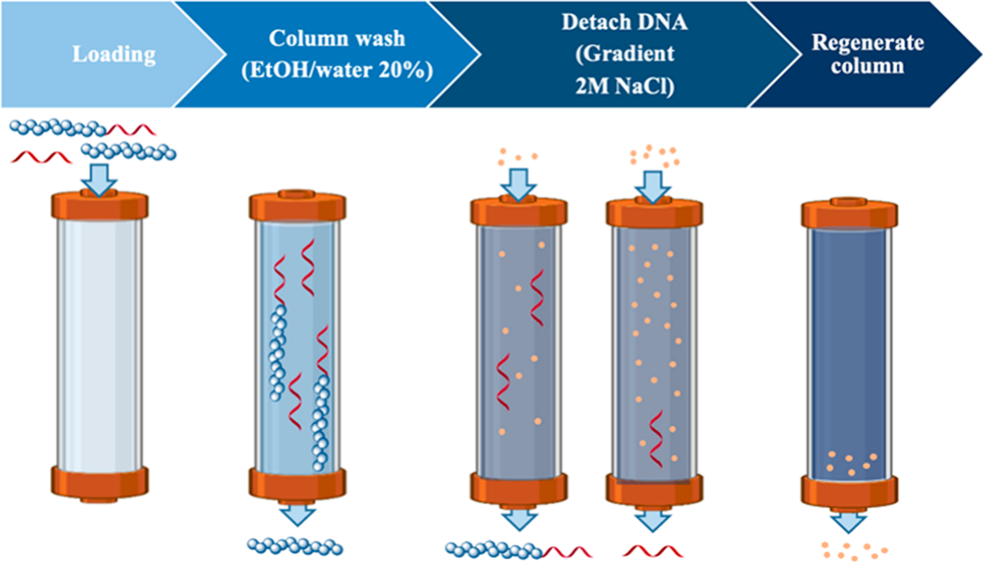

We present a facile and adaptable method to purify and isolate DNA–polymer conjugates from different uncharged homo, random, or block copolymer families. Anion exchange chromatography is used to separate the reaction solution and retrieve the excess unreacted polymer and oligonucleotide. The stationary phase has a high efficiency (25 nmol of DNA per run), facilitating the purification of large batches without compromising the peak shape and resolution. To demonstrate the versatility of this method, different types of polymers, including acrylates, methacrylates, and acrylamides containing hydrophilic and hydrophobic blocks, were purified with high yields. Additionally, DNA–polymer conjugates with various DNA block lengths were also successfully purified, further highlighting the broad applicability of this method.

DNA–polymer conjugates have seen a significant expansion in recent years due to the increasing accessibility of DNA, resulting in seminal developments ranging from gene therapy^1,2^ to drug delivery^3−6^ and biosensing.^7−9^ Exploiting the fidelity of DNA technology, they can be used to construct sophisticated shapes and patterns that are only a few nanometers in size, leading to new possibilities for the design and construction of precision nanoscale devices.^10,11^ Furthermore, functional polymer or polymer-coated DNA architectures lead to an enhanced cellular uptake, thereby increasing pharmaceutical applications, for instance as drug carriers.^12−14^ Additionally, by varying the length and composition, they can provide diverse architectures including micelles, vesicles, and tubes.^15^ The individual control over the polymer and DNA length, composition, and chemical handles enables broad engineering of conjugate properties for targeted applications.^14,16^

To prepare DNA–polymer conjugates, the *grafting-to* method is commonly used, which involves using excess amounts of the less expensive polymer to achieve high conversion rates.^10,17−19^ However, this often leads to the challenge of removing the unreacted polymer after the reaction. Because of the high amount of free polymer and the amphiphilic nature of the conjugates, spin filtration with molecular weight cutoffs often results in long purification times and major product loss.^10^ Other methods such as reversed-phase high performance liquid chromatography (HPLC) easily reach their capacity limits, requiring extensive optimization of the stationary and mobile phases according to different polymer scaffolds and molecular weights. Alternatively, size exclusion chromatography (SEC) is particularly inefficient for smaller DNA blocks and the often amphiphilic nature of the conjugates.^20^ As such, the full integration of polymer science into DNA nanotechnology has not been fully realized due to these pervasive challenges.

To address this limitation, we expand existing approaches of purifying DNA–polymer conjugates with strong anion exchange chromatography,^21,22^ explore its broad applicability toward DNA lengths, different polymer scaffolds, and molecular weights, and compare it with other techniques like HPLC, SEC, and spin filtration. The strategy of this method relies on the negative charge of the DNA block to interact with the positively charged stationary phase. Excess uncharged polymer moves freely through the column and is eluted. Subsequent change in the mobile phase with a gradient of NaCl solution is used to elute DNA containing molecules and allows separation of the DNA–polymer conjugate from minor amounts of unreacted oligonucleotide. Within this method, polymers of three different polymer families (acrylates, acrylamides, and methacrylates) with molecular weight between 9 and 48 kDa were purified as well as conjugates with different DNA block lengths (10, 19, and 40 bases).

Adopting the *grafting-to* strategy, the polymer block is synthesized using polymerization techniques via reversible addition–fragmentation chain transfer (RAFT), which incorporates a functional end-group. Subsequently, the polymer is covalently attached to a complementary functional group on the DNA block. In this case, the strategy was performed using NH_2_-functionalized oligonucleotide and NHS-activated polymers prepared by RAFT polymerization.^10^ The coupling was performed in a DMF/water (3:1) mixture using 50 equiv of the polymer, with DIPEA as an auxiliary base (Figure 1b). The reaction solution was analyzed using polyacrylamide gel electrophoresis (PAGE) to confirm the formation of the DNA–polymer conjugate and to monitor remaining oligonucleotides (Figures 1d and S22). Using the general conditions above, polymers (P(DMA), **P1**, 9.6 kDa; P(DMA), **P2**, 22 kDa; P(DMA), **P3**, 48 kDa; P(NIPAM), **P4**, 21 kDa; P(OEGMA), **P5**, 21 kDa; P(DAAM-*b*-DMA), **P6**, 26 kDa; P(HEA), **P7**, 22 kDa; P(DAAM-*co*-DMA) **P8**, 26 kDa; P(NIPAM-*b*-DMA) **P9**, 30 kDa) and DNA of various lengths (**SDNA**, 10 base long; **DNA**, 19 base long; **LDNA**, 40 base long) were prepared and coupled. P(DMA) (**P1–P3**) with varying molecular weights was selected to establish a reliable method and to investigate the impact of polymer block length on the conjugation efficiency. P(NIPAM) (**P4**) and P(HEA) (**P7**) were employed to demonstrate how changes in the properties of the polymer block, specifically hydrophilicity and hydrophobicity, affect the method. The brush-like P(OEGMA) (**P5**) represents a sterically demanding polymer which potentially influences the binding to the stationary phase. Block copolymers P(DAAM-*b*-DMA) (**P6**) and P(NIPAM-*b*-DMA) (**P9**) as well as random copolymer P(DAAM-*co*-DMA) (**P8**) were also included to demonstrate the robustness of the technique toward complex polymer designs. Additionally, DNA block lengths were varied to show the ease of method adaptation caused by charge and differences in hydrophilicity.

**Figure 1:**
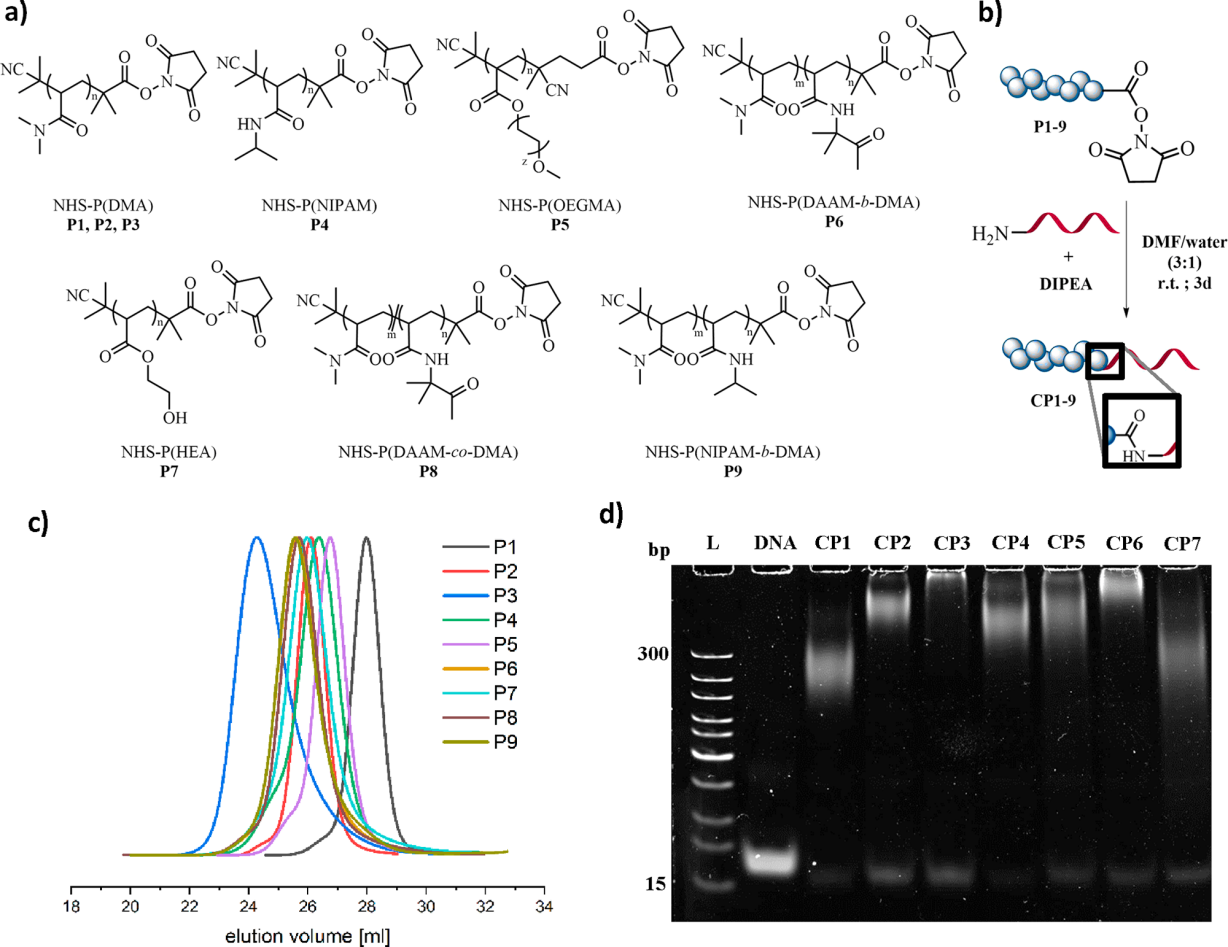
(a) Chemical structure of the NHS polymers used for coupling with the respective DNA. Synthesized via RAFT. (b) General reaction scheme of the NHS coupling to an amine-functionalized oligonucleotide. (c) Elution diagrams of the NHS polymers **P1**–**P9** as measured by DMF SEC using poly(methyl methacrylate) (PMMA) as standard. (d) PAGE gel of the obtained reaction solutions after coupling **P1**–**P7** to 19-base-long oligonucleotide. L: DNA ladder; DNA: used 19-base-long oligonucleotide; **CP1**–**CP7** respective coupling reaction solution of **P1**–**P7**. Using SYBR Gold for staining (2×).

Following polymer to DNA coupling, DMF and DIPEA were removed via spin filtration, and the obtained solution was diluted with water to a final volume of 2 mL, from which 1 mL (25 nmol of DNA) is loaded onto the column (Cytiva HiRes Q 5/50; 5 × 50 mm^2^ bed dimensions). The purification method contains several steps to prepare/equilibrate the column, separate the components of the reaction solution, and elute them fractionwise (Figure S1). Detection at 260 nm during purification takes into consideration the characteristic absorption of DNA.^23^ Additionally, as the polymer absorbs mostly at 240 nm, it is also tracked simultaneously (Figure S2). To prepare the column, the stationary phase is initially washed and equilibrated with water (flow rate: 0.7 mL/min; 5 CV; Figure S3) before loading with the diluted reaction solution (Figure S4). Both DNA and the DNA–polymer conjugate are negatively charged, allowing the molecules to bind to the positively charged resin.^24^ The sample loading is achieved via a 1 mL sample loop and applying 3 mL of water as the loading volume. Subsequently, the column is washed with EtOH/water (20% EtOH, v/v, 20 CV) to remove the unbound, excess free polymer from the solution (Figure S5). Next, stepwise elution is performed by applying a NaCl gradient to control the separation of the bound components (Figure S6). Post elution, the column is washed with 2 M NaCl solution to remove any remaining bound components (Figure S7). Finally, the stationary phase is equilibrated with pure water, and a new run can begin (Figure S8). Because of the charged resin’s high binding affinity towards DNA, 25 nmol of DNA per run can be purified without a loss of resolution or purity of the conjugate, despite the small size of the column.

The versatility of the method was demonstrated using NHS-functionalized polymers from three different monomer families (acrylates, methacrylates, and acrylamides) coupled to the 19-base oligonucleotide (Figure 1a). First, using DMA as the scaffold, the polymer length of P(DMA) was varied, comprising a short (P(DMA) ∼9.6 kDa; **P1**), middle (P(DMA) ∼22 kDa; **P2**), and a longer (P(DMA) ∼48 kDa; **P3**) chain to test if the purification remained robust. The elution diagram for the DNA–polymer conjugate **CP2** (Figure 2a) indicates that during loading and washing most of the polymer was successfully removed from the column. Subsequently, the eluent was changed to a gradient of NaCl (0–2 M) solution. As the NaCl solution concentration increases from 0 to 0.4 M, further unbound polymer was eluted from the column. At 0.4 M NaCl, the mobile phase was held isocratic to elute the DNA–polymer conjugate followed by the unreacted DNA oligonucleotide. Thereafter, gradient elution from 0.4 to 2 M NaCl was implemented to regenerate the stationary phase, removing any remaining bound components. Surprisingly, the elution diagrams (Figures 2a and S9–S10) showed no significant changes during purification of the corresponding DNA–polymer conjugates **CP1**–**CP3** (elution volumes: CP1, 31.3 mL; CP2, 31.0 mL; CP3, 30.3 mL). However, the trend shows that the larger the polymer block, the faster the conjugate elutes. We postulate that as the polymer segment increase in size, its collapsed volume increases correspondingly, which affects the accessibility to the DNA block and lowers the binding affinity to the stationary phase. Characterization of the purified DNA–P(DMA) conjugates via PAGE (Figure 2b) and SEC (Figure 2c) demonstrates the successful isolation. The respective yields range from ∼67% to quantitative (Table S2).

**Figure 2:**
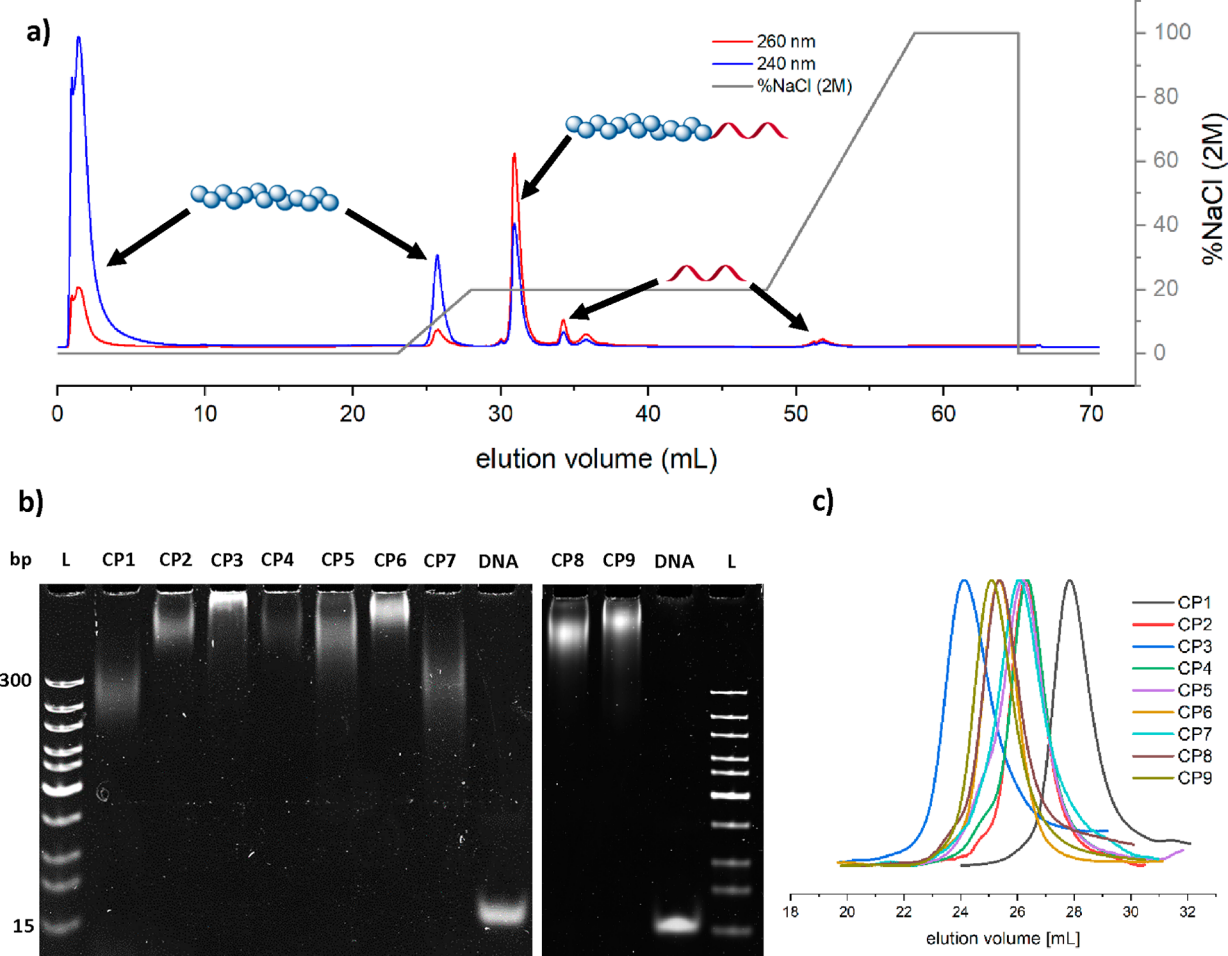
(a) Elution diagram of the **CP2** reaction solution run with contemporaneous detection of absorbance at 240 nm (polymer block) and 260 nm (oligonucleotide block). (b) PAGE gel electrophoresis of the respective purified conjugates **CP1**–**CP9**. L: DNA ladder; **CP1**–**CP9**: respective pure conjugates; DNA: 19-base oligonucleotide as comparison. Stained with SYBR Gold (2×). (c) Elution diagrams of the conjugates **CP1**–**CP9** measured by DMF SEC using poly(methyl methacrylate) (PMMA) as standard.

Subsequently, the methodology was applied to a broader range of polymer species, including the more hydrophilic poly(hydroxyl acrylate) (P(HEA), ∼22 kDa; **P7**) and the more hydrophobic and thermoresponsive^25−27^ poly(*N*-isopropylacrylamide) (P(NIPAM), ∼21 kDa; **P4**). Despite the varying hydrophilic character of the polymer block, the elution profiles of **CP4** and **CP7** had no significant impact on the separation efficiency (elution volume: **CP4**: 31.5 mL, Figure S11; **CP7**: 31.8 mL, Figure S14). Good to excellent yields were obtained for **CP4** (∼98%) and **CP7** (∼70%). The next attempt was to purify more sterically demanding DNA–polymer conjugates by using poly(oligo(ethylene glycol) methacrylate) (P(OEGMA), ∼21 kDa; **P5**). The elution profile remained unaffected during the purification process (Figure S12). The elution volume of **CP5** is 30.7 mL, with a yield of ∼63% (Table S2). The excess of unreacted brush-like polymer could also be eliminated, as confirmed by SEC (Figure 2c).

The established method was further tested with an amphiphilic block copolymer, which is known to undergo partial phase separation.^28^ A diacetone acrylamide–DMA block copolymer (P(DAAM-*b*-DMA), ∼26 kDa; **P6**) and a *N*-isopropylacrylamide–DMA block copolymer (P(NIPAM-*b*-DMA), ∼30 kDa; **P9**) were synthesized, coupled to DNA to afford **CP6** and **CP9**, and purified using the proposed method. Consistent with the other polymer species tested, no major changes were noted during the purification step, as the elution volume is 31.1 mL for **CP6** (Figure S13) and 31.0 mL for **CP9** (Figure S17). An isolated yield of ∼60% for **CP6** and ∼72% for **CP9** was obtained, which was similar to that of the brush-like **CP5** conjugate. For comparative purposes, a random diacetone acrylamide–DMA copolymer (P(DAAM-*co*-DMA ∼26 kDa, **P8)** was subjected to coupling and purified, giving an elution time for **CP8** of 31.6 mL (Figure S16) and 93% yield. This enhanced yield could be attributed to the reduced phase separation of the polymer during the purification process. These results indicate the method’s universal applicability for a broad range of water-soluble DNA/polymer conjugates with uncharged polymer segments.

Although the variation in polymer lengths and composition had a minimal impact on the chromatographic separation, the length of the DNA segment contributes to the total charge and should therefore influence the elution profile. To this end, we selected 10-base (NH_2_-CCACCTACTA; **SDNA**) and 40-base (NH_2_-GAAGATAAAAACATTTGATTTTTTCTCTACCACCTACTA; **LDNA**) oligonucleotides in addition to the 19-base counterpart (Figure 3a). With the different oligonucleotides, polymer **P2** was used as the model polymer and coupled to the DNA strand via the same NHS chemistry. As an initial assessment, PAGE confirmed the successful reactions (Figure 4a). For the purification of the long DNA–polymer conjugate, we used the same gradient optimized for the 19-base oligonucleotide (Figure S15). However, unlike the 19-base DNA–polymer conjugates, the 40-base counterpart (**LCP2**) eluted at a high retention volume (51–53 mL) together with the unreacted oligonucleotide at higher NaCl concentrations (0.85–1.2 M). Hence, the gradient was modified in order to resolve the separation. We developed a stepwise gradient method to identify the optimal NaCl concentration for elution (Figure 3b). The individual peak fractions were collected, and PAGE was performed to identify the components (Figure 3c). We found that 0.5 M NaCl was the optimal concentration to selectively elute the conjugate, **LCP2**. Using this knowledge, the original elution diagram was modified with a gradient run until 0.5 M, which was then held isocratic to successfully separate the conjugate **LCP2** from the free oligonucleotide (Figure 4b). Because of the gradient adjustment, which was necessary for **LCP2**, we applied a similar approach for **SCP2** by reducing the gradient to 0.3 M NaCl (Figure 4d), resulting in pure conjugate as verified via PAGE gel analysis (Figure 4e). This experiment series demonstrates the method’s ease and flexibility in purifying DNA–polymer conjugates with varying DNA block lengths commonly used within DNA nanotechnology. Based on straightforward method development protocols, further variation of the DNA block lengths can be easily resolved by the NaCl gradient during elution.

**Figure 3:**
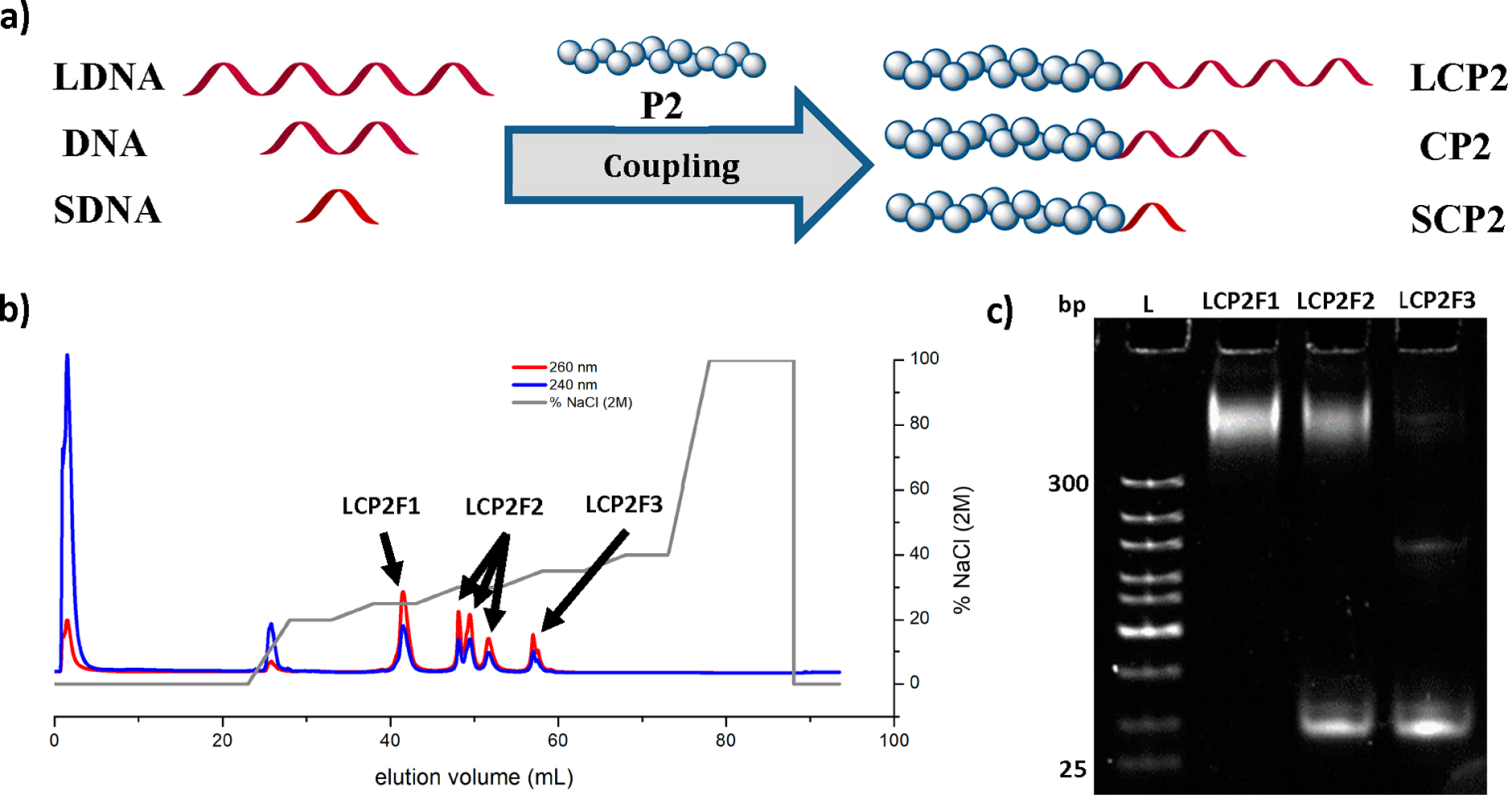
(a) Schematic representation of the coupling reaction of the generated NHS-P(DMA) (**P2**) to the oligonucleotides of different lengths (10, 19, and 40 bases). (b) Elution diagram of the method development profile of the **LCP2** reaction solution to find the optimal gradient for the conjugate elution. Here the gradient, starting at 20% was every 5 CV increased by 5% to 40%. (c) PAGE of the **LCP2** test run fractions as shown in b). L: DNA ladder; **LCP2F1–3**: respective fractions. Stained with SYBR Gold (2×).

**Figure 4:**
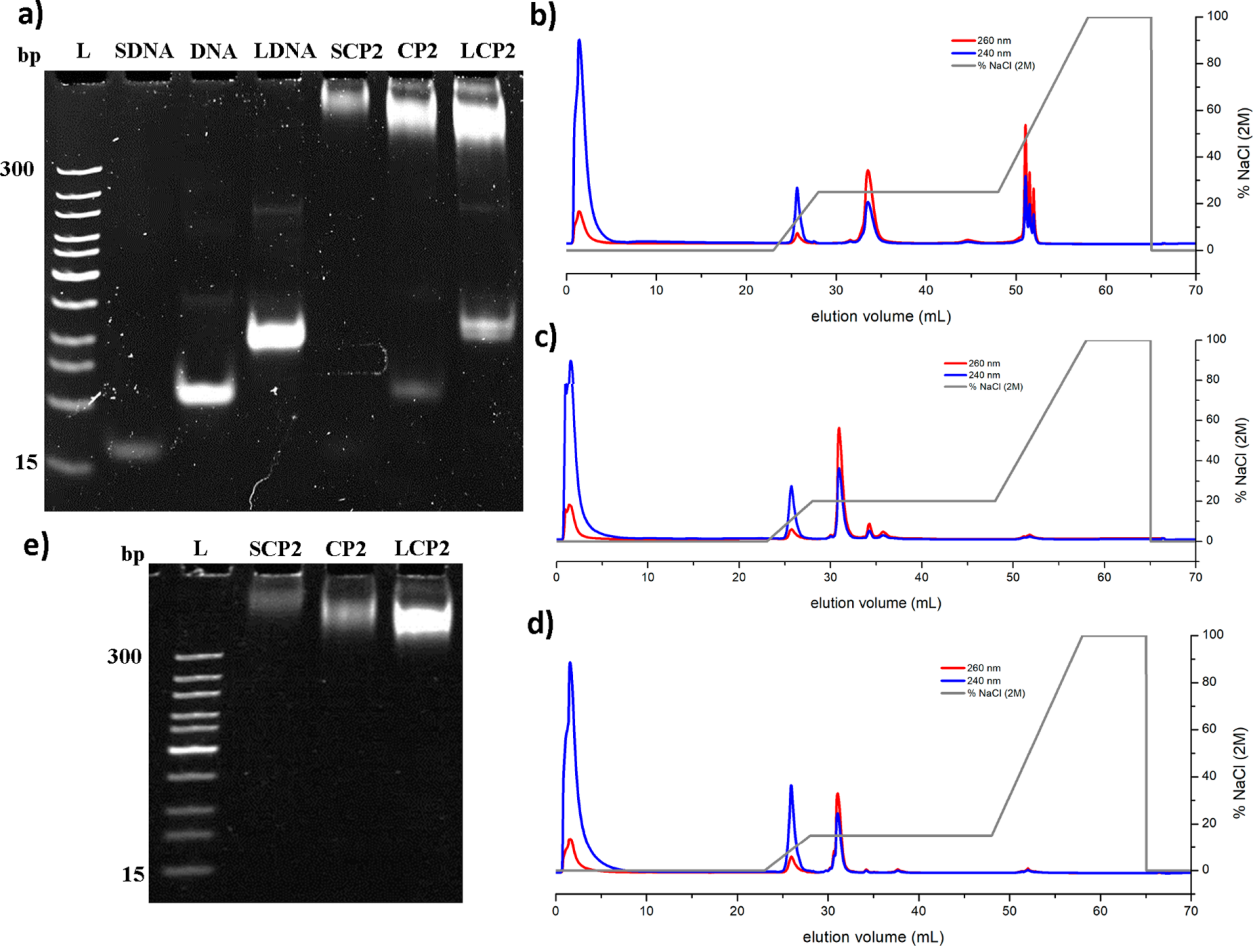
(a) PAGE gel electrophoresis of the pure oligonucleotides and of the reaction solutions after coupling with **P2**. L: DNA ladder; **SDNA**: short oligonucleotide (10 base long); **DNA**: middle oligonucleotide (19 base long); **LDNA**: long oligonucleotide (40 base long); **SCP2**: reaction solution of **P2** coupled with **SDNA**; **CP2**: reaction solution of **P2** coupled with DNA; **LCP2**: reaction solution of **P2** coupled with **LDNA**. Stained with SYBR Gold (2×). (b–d) Elution diagram of the whole purification run of the coupling reactions of **LCP2**, **CP2**, and **SCP2** with **P2** with contemporaneous detection of absorbance at 240 nm (polymer block) and 260 nm (oligonucleotide block). (b) **LDNA** reaction solution. (c) **DNA** reaction solution. (d) **SDNA** reaction solution. (e) PAGE gel electrophoresis of the obtained pure conjugates of **SCP2**, **CP2**, and **LCP2**. L: DNA ladder; **SCP2**/**CP2**/**LCP2**: pure conjugate of the respective conjugate was obtained after purification. Stained with SYBR Gold (2×).

Using **CP2** as a model DNA–polymer conjugate, we compare our method against analytical scale HPLC and SEC as well as with spin filtration alone. The HPLC studies (Jupiter C18, 5 μm, 300 Å) were performed first using the reaction mixture of **CP2** (8 μL, 1 nmol), containing 75% (v/v) DMF. Stepwise gradient of acetonitrile/water elution shows several peaks divided into five fractions (F1: 1.25–3.75 mL; F2: 16–16.8 mL; F3: 16.8–17.8 mL; F4: 19–21 mL; F5: 22–22.6 mL) (Figure S18d,e). PAGE gel (Figure S18f) revealed an incomplete separation of the unreacted oligonucleotide and **CP2**. While F1 shows no conjugate or DNA, F2–F4 include **CP2** as well as unreacted oligonucleotide, whereas F5 contains only **CP2**. Intensity measurements of the bands revealed a yield of 13% for F5 (Table S5). Removal of DMF by spin filtration prior to HPLC separation did not improve the peak resolution (Figure S19). However, the yields of pure fractions F3 and F4 were increased to 45% (Table S5). These results show that even in an analytical scale, purification of **CP2** and separation from unreacted oligonucleotide are not optimal. Separate attempts using analytical SEC performed with 8 μL (1 nmol) reaction solution of CP2 fail to show visible separation due to the large excess of polymer (50 equiv) present (Figure S20). Purification solely by the spin filtration method (8 cycles) with 10 and 30 kDa molecular weight cutoff were similarly unsuccessful. In each case, the PAGE gel (Figure S21) indicated the continued presence of free oligonucleotide after purification. Overall, these methods present very limited success in isolating pure DNA–polymer conjugates.

The purification efficiency of the method relies on the accessibility of the DNA block to the anionic stationary phase, which is in turn dictated by the length of the polymer block and the steric component of the side-chains. Given a fixed oligonucleotide length of 19 bases, purification efficiency decreases with increasing polymer length (yields: **CP1** (∼9.6 kDa, quantitative), **CP2** (∼22 kDa, 96%), **CP3** (∼48 kDa, 67%)). Second, polymers with side-chain bulk such as that of P(OEGMA), **CP5**, or amphiphilic block copolymers like **CP6** reduced the ionic interactions between the oligonucleotide to the column, which in turn lowered the yields. In general, polymers with a molecular weight between 10 and 50 kDa with small, homogeneous side-chains are ideal for this purification method.

In conclusion, we have introduced a versatile and adaptable purification method with high efficiency to purify DNA–polymer conjugates containing various classes of polymers (acrylates, methacrylates, and acrylamides) and oligonucleotide lengths. Compared to other performed methods like HPLC, spin filtration, or SEC, anion exchange chromatography consistently provides high yields in preparative scale. The purification method is largely independent of the polymer, and even sterically demanding, brush-like DNA–polymer conjugates were isolated successfully. Moreover, complex DNA–polymer conjugates like DNA–P(DAAM-*b*-DMA) and P(NIPAM-*b*-DMA), with a high tendency to phase separate, could also be purified without loss in resolution. The purification of DNA blocks of different lengths is easily adaptable, which will widen the range of applications of this method in the community. As the unreacted oligonucleotide can also be separated, reuse would also be possible. This reported approach is reliable for water-soluble, noncharged polymer block within DNA–polymer conjugates and can be scaled according to the column dimensions holding the stationary phase. The increased ease of purification and isolation of DNA–polymer conjugates will create greater access and application in nanoscale engineering, biomedicine, and polymer science.

## Supporting Information Available

Detailed information about the polymer synthesis, the coupling reactions, and the purification method (PDF)
