# Supplementary material for: A Versatile and Efficient Method to Isolate DNA–Polymer Conjugates
Source: ACS Macro Lett. 2023 Sep 1;12(9):1257–63. doi: 10.1021/acsmacrolett.3c00371 (PMC10515633; doi:10.1021/acsmacrolett.3c00371)
Supplement: Supplementary file 1 — mz3c00371_si_001.pdf [file mz3c00371_si_001.pdf]

Electronic Supplementary Information

A Versatile and Efficient Method to Isolate DNA-Polymer Conjugates

Nico Alleva, Katharina Eigen, David Y. W. Ng, Tanja Weil

Corresponding Author:

E-Mail: David Ng david.ng@mpip-mainz.mpg.de

Tanja Weil weil@mpip-mainz.mpg.de

Max Planck Institute for Polymer Research, Mainz 55128, Germany

CHEMICALS

Diethyl ether (Honeywell), petrolium ether (30-40 °C, Fisher Scientific), dichloromethane (Fisher Scientific),Tetramethylethylenediamine (TEMED) (Roth, >98.5%, p a), GeneRuler Ultra Low Range DNA Ladder (Thermofisher), tris-borate-EDTA buffer (Sigma Aldrich, 10x concentrate), nuclease-free water (QIAGEN), *N,N*-diisopropylethylamine (Roth, >99,5%), 1,4-dioxane (Sigma Aldrich, anhydrous, 99.8%), dimethylformamide (ACROS, extra dry, 99.8%), 4-cyano-4-(phenylcarbonothioylthio)pentanoic acid *N*-succinimidyl ester (Sigma Aldrich) and 2-(dodecylthiocarbonothioylthio)-2-methylpropionic acid *N*-hydroxysuccinimide ester (Sigma Aldrich) were used as received.

2,2'-Azobis(2-methylpropionitrile) (Fluka analytics, >98%) was recrystallized in methanol prior use.

Poly(ethylene glycol) methyl ether methacrylate (PEGMA, Sigma Aldrich, average M_n = 300 g/mol, stabilized with 100 ppm MeHQ and 300 ppm BHT), *N,N*-dimethylacrylamide (DMA, Sigma Aldrich, 99%, stabilized with 500 ppm MeHQ) and 2-hydroxyethyl acrylate (HEA, Acros, 97%, stabilized) were purified prior polymerization by removing the stabiliser with a small column filled with alumina.

N-Isopropyl acrylamide (NIPAM, TCI, >98%, stabilized with MeHQ) and diacetone acrylamide (DAAM, Alfa Aesar, 99%,) were purified by dissolving in dioxan and removing the stabiliser with a small column filled with alumina.

PROCEDURES AND METHODS

POLYMERIZATION

The polymerization procedure was carried out as described by Alleva et al. 2022[1]

For the polymerization of homo polymers or the first block of the block copolymers, the monomer, CTA and AIBN were dissolved in the polymerization solvent, purged with argon for 45-90 min and heated up to the respective temperature. The ratio of initiator to CTA was 1:10. After the reaction time, the reaction mixture was cooled down with an ice bath and a precipitate was formed in the respective precipitation solvent (Table S1). The collected solid was again dissolved and precipitated twice. The obtained solid was dried under vacuum.

The second block was obtained by dissolving the first block (macro CTA) in the polymerization solvent, adding AIBN and monomer, purged with argon for 45-90 min and heated up to respective temperature. After the reaction time, the reaction solution was cooled with an ice bath and precipitated in the precipitation solvent. The obtained solid was collected and dried under vacuum.

CTA REMOVAL

The polymerization procedure was carried out as described by Alleva et al. 2022[1]

The obtained polymer was dissolved in dioxane and an excess of AIBN was added. The reaction solution was heated up to 80 °C. After the reaction time, the reaction solution was cooled in an ice bath, and the polymer was participated in the respective precipitation solvent (Table S1). The obtained polymer was dried under vacuum and analysed with SEC (DMF, PMMA standard).

CONJUGATION REACTION

For a typical conjugation reaction, oligonucleotide (SDNA: NH_2-CCACCTACTA ; DNA: NH_2-TTTTCTCTACCACCTACTA or LDNA; NH_2-AGAAGATAAAAACATTTGATTTTTTCTCTACCACCTACTA) (50 nmol), polymer (50 equiv.) and DIPEA (200 equiv.) were mixed in a DMF/water (3:1) mixture to a total volume of 400 µL (125 µM DNA) and shaken for at least 48 h at room temperature. After the reaction time, 2.5 µL of the reaction solution was diluted and analysed with PAGE.

PAGE

The PAGE gel (15%) was prepared by mixing tetramethylethylenediamine (TEMED) (7.5 µL), 40% acrylamide/bis-acrylamide solution 37.5:1 (5.63 mL), 10x TRIS-borate-EDTA buffer (TBE buffer) (1.5 mL), water (7.9 mL) and 10% ammonium persulfate (APS) solution (75 µL) and casting the gel.

For monitoring the conjugation reaction via PAGE, 1 µL of diluted reaction solution (25 pmol DNA, for SDNA 62.5 pmol due to its smaller size and less intercalation of the dye) was mixed with water and loading dye (1.7 µL, 6x Thermo Fisher) to a total

volume of 10 µL and loaded onto the gel. The gel was run first at 100 V for 10 min and then at 150 V for 45–50 min on a Cell SureLockTM mini-cell electrophoresis system from Thermo Fisher using 0.5 × TBE buffer as the running buffer (44.5 mM Tris-Borate, 1 mM EDTA). Gene ruler ultra-low range DNA ladder (Thermo Fisher) was used as the DNA ladder. The gels are stained with SYBR Gold (2×, 50 mL) for 45 min at room temperature. The images were taken with ChemiDoc Touch Imaging System from Bio-Rad or G:BOX Chemi Gel Doc System from Syngene. For intensity measurement ImageJ was used to determine the intensity of the respective band.

SEC

Polymer measured with SEC: 10–20 mg of dry polymer was measured via the SEC method stated below.

Preparing the obtained DNA-polymer conjugate for SEC: 100–200 µL of the obtained conjugate solution was freeze dried. The obtained solid was measured via the SEC method stated below.

For the purification approach, 8 µL (1 nmol) were diluted with DMF to 100 µL and injected.

SEC experiments were performed on a PSS SECurity instrument comprising an auto sampler, a column oven with three GRAM columns (10^3, 10^3 and 10^2 Å, 300 × 8 mm, 10 µm particle size) and a RI as well as an UV detector (Agilent Technologies 1260 Infinity). DMF containing 1 g/L lithium bromide was used as the eluent at a flowrate of 1 mL/min. Poly(methyl methacrylate) (1600 kDa–800 Da) served as the calibration standard for molecular weight measurements. The samples were filtered (0.4 µm) prior to injection. The data were fitted with OriginPro 2021.

Spin filtration (sF) for purification

For the spin filtration, 200 µL of reaction solution, containing 25 nmol DNA were given to the respective spinfilter ((Amicon Ultra-6 mL Centrifugal Filters MWCO 10k or 30k cutoff) and were centrifuged 8 times for 1 hour. After each centrifugation the filtrate was discarded, the filter was refilled with 6 mL water and the solution was mixed. Centrifuged at 3900 rpm.

High pressure liquid chromatography (HPLC) for purification

HPLC instrument from Shimadzu was used including an auto sampler, a column oven and a fraction collector. The samples were purified by analytical HPLC using the column Jupiter 5 µm C18 300A (4.6 × 150 mm, 5 µm at a flowrate of 1 mL/min. The elution protocol started with the mobile phase from 5% solvent B (HPLC grade acetonitrile) and 95% solvent C (0.1 M triethylammonium acetate buffer), raising to 15% B, then to 25% B and then increasing to 100% B, hold it for 20 min then decreasing to 5% B in 4 min. The absorbance was monitored at 240 nm and 260 nm. 8 µL (1 nmol) were injected.

Tecan absorbance measurement

To determine the concentration of the obtained DNA–polymer conjugate solutions after the purification, Tecan Spark was used to measure the absorbance at 260 nm. Extinction coefficient of the SDNA block: 83720 $L \cdot mol^{-1} \cdot cm^{-1}$ DNA block: 187700 $L \cdot mol^{-1} \cdot cm^{-1}$ LDNA block: 344186 $L \cdot mol^{-1} \cdot cm^{-1}$; d = 0.5 mm.

Anion exchange column

Cytiva Capto™ HiRes Q 5/50 was used for the purification of DNA–polymer conjugates. The column was stored at 20% EtOH/water.

ÄKTA system

The ÄKTA pure™ system was used as the device for using the anion exchange column.

Preparing reaction solution for AKTA purification

The included DMF of the obtained reaction solutions were removed via spin filtration (Amicon Ultra-6 mL Centrifugal Filters MWCO 2k or 10k) by adding 5 mL nuclease-free water to the reaction solution and centrifuge for 1 h. This was repeated three times.

ÄKTA PURIFICATION METHOD

The following pictures/screenshots show the purification method, used with the ÄKTA pure™ with the anion exchange column Cytiva Capto™ HiRes Q 5/50 build in. The screenshots show the method applied for all conjugate purifications containing the 19 base oligonucleotide. The gradient is driven to 20% and held then isocratic. Please notice that for the short 10 base oligonucleotide the gradient is driven to 15% instead of 20% and for the long 40 base oligonucleotide the gradient is driven to 25%. All other parameters, pressures, flow rates etc. are the same.

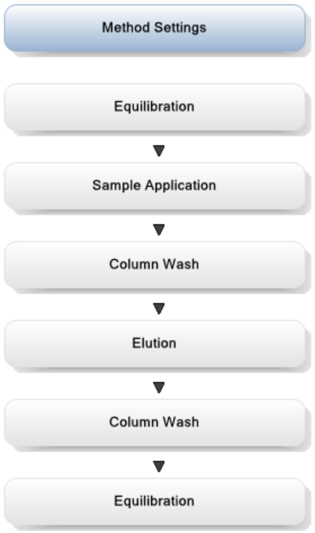

Figure S1: Overview of the single steps of the purification method

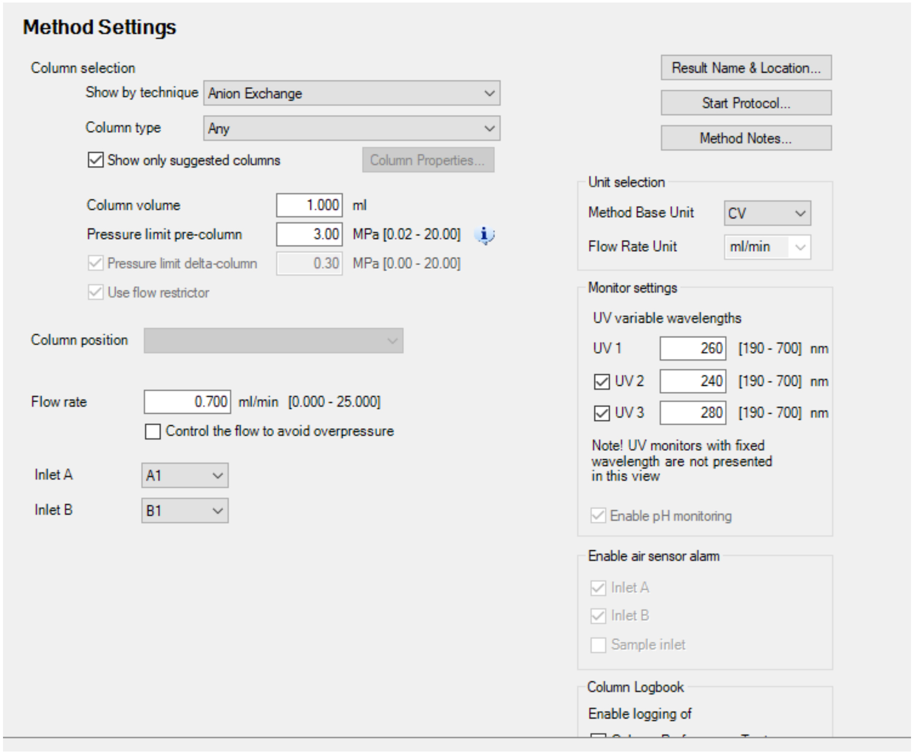

Figure S2: General method settings. A1 is water and B1 is NaCl solution (2M).

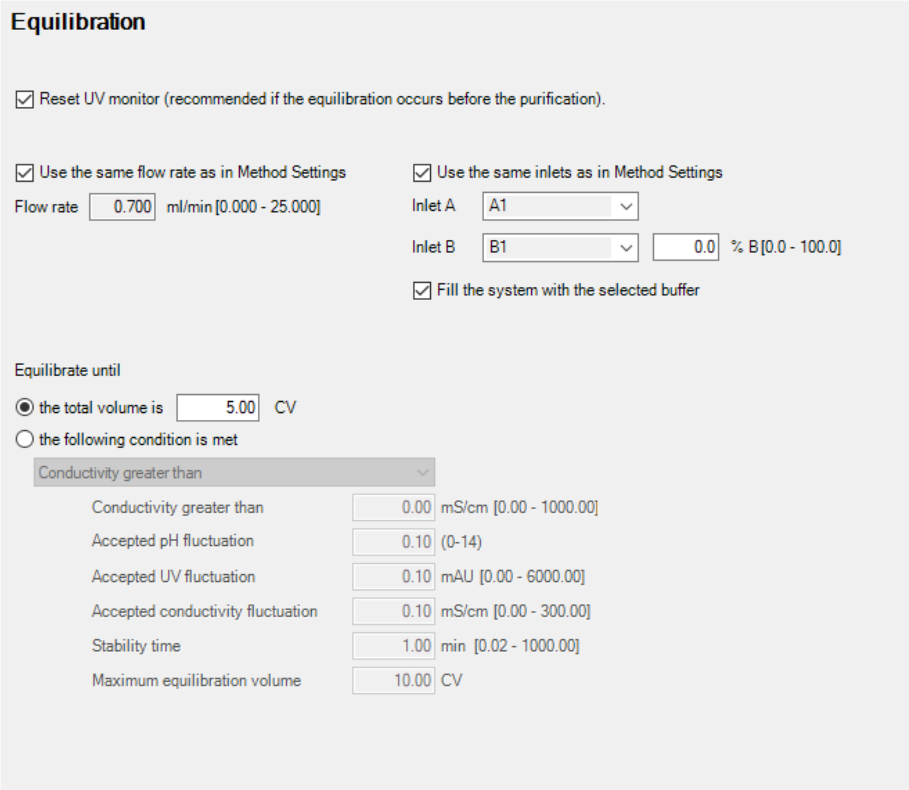

Figure S3: Equilibration step.

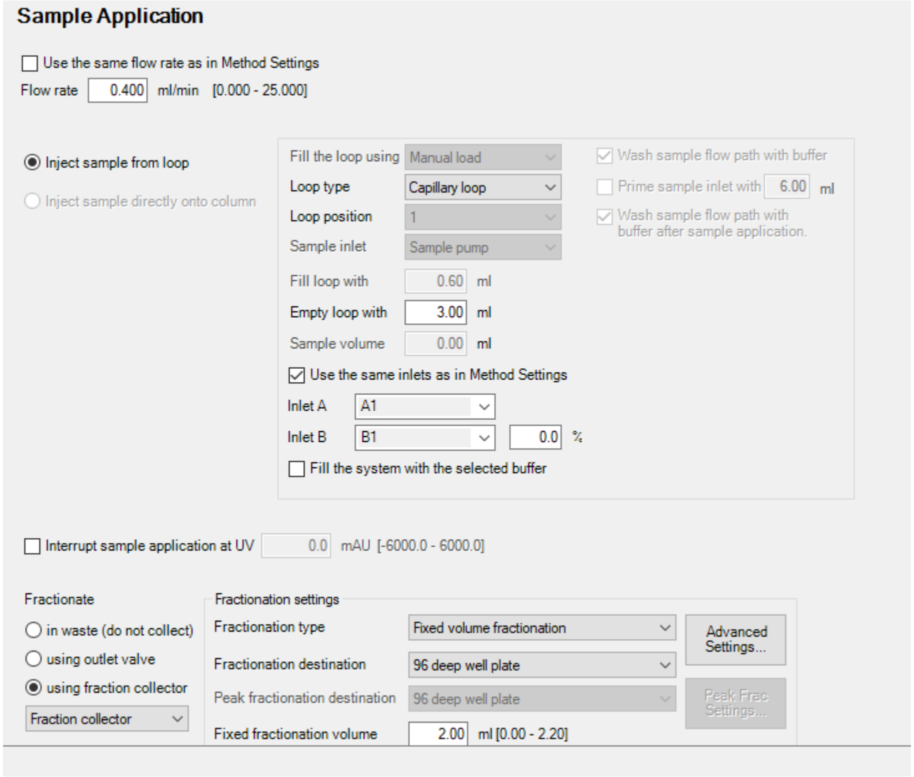

Figure S4: Sample application step.

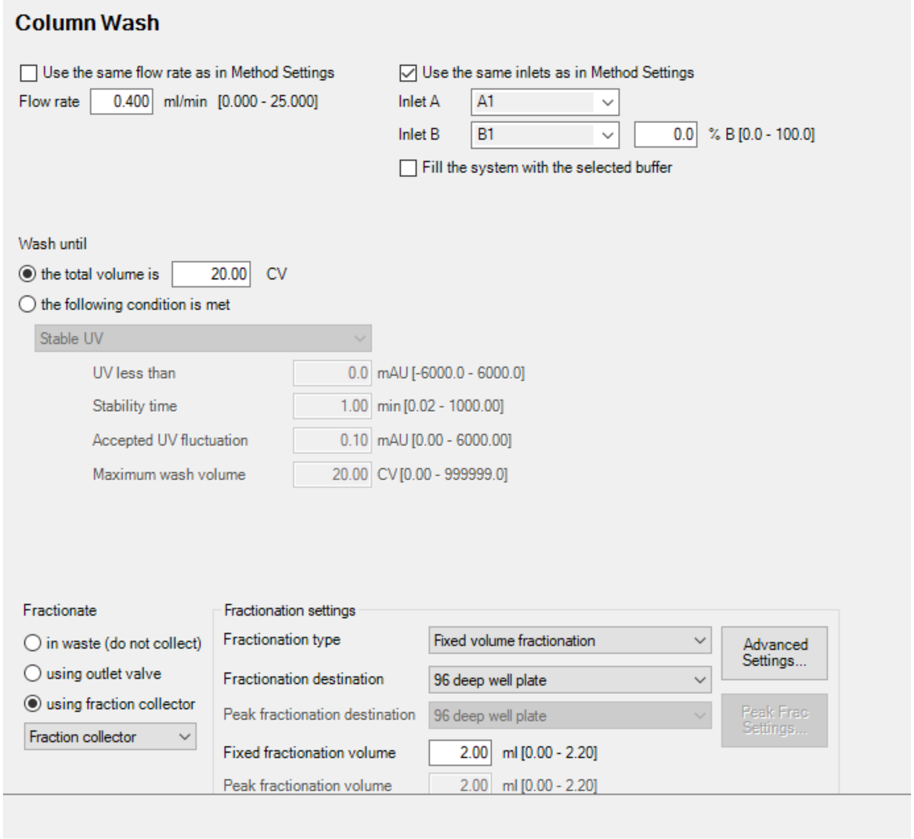

Figure S5: First column wash step to remove the uncharged, unreacted polymer excess.

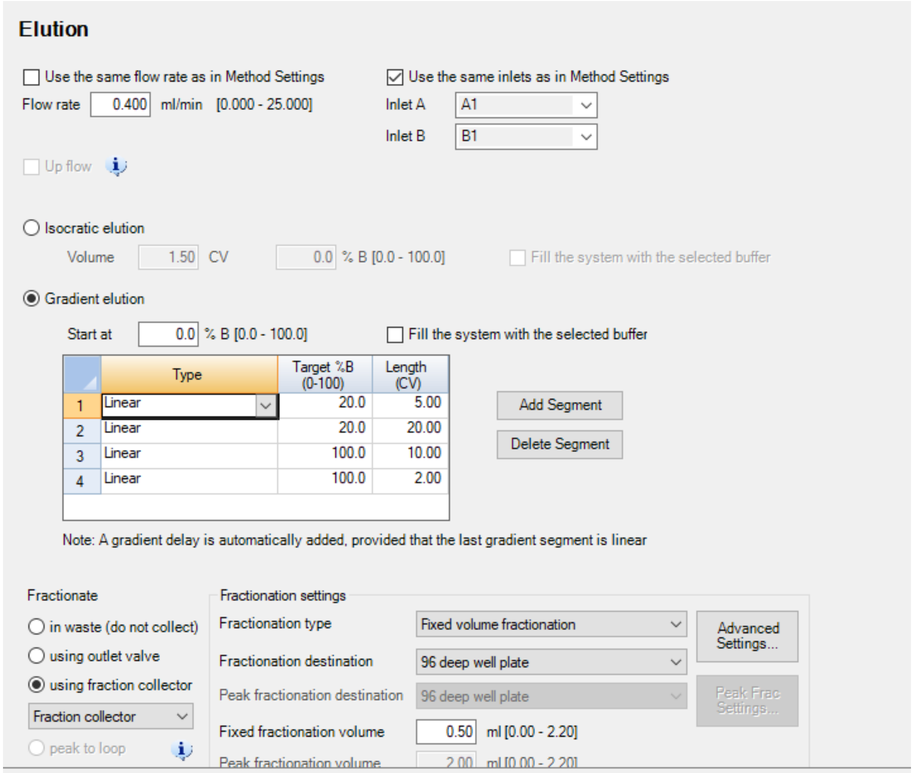

Figure S6: Elution step. In this step the gradient is driven to the respective limit to elute the DNA-polymer conjugate and at higher concentrations the unreacted oligonucleotide.

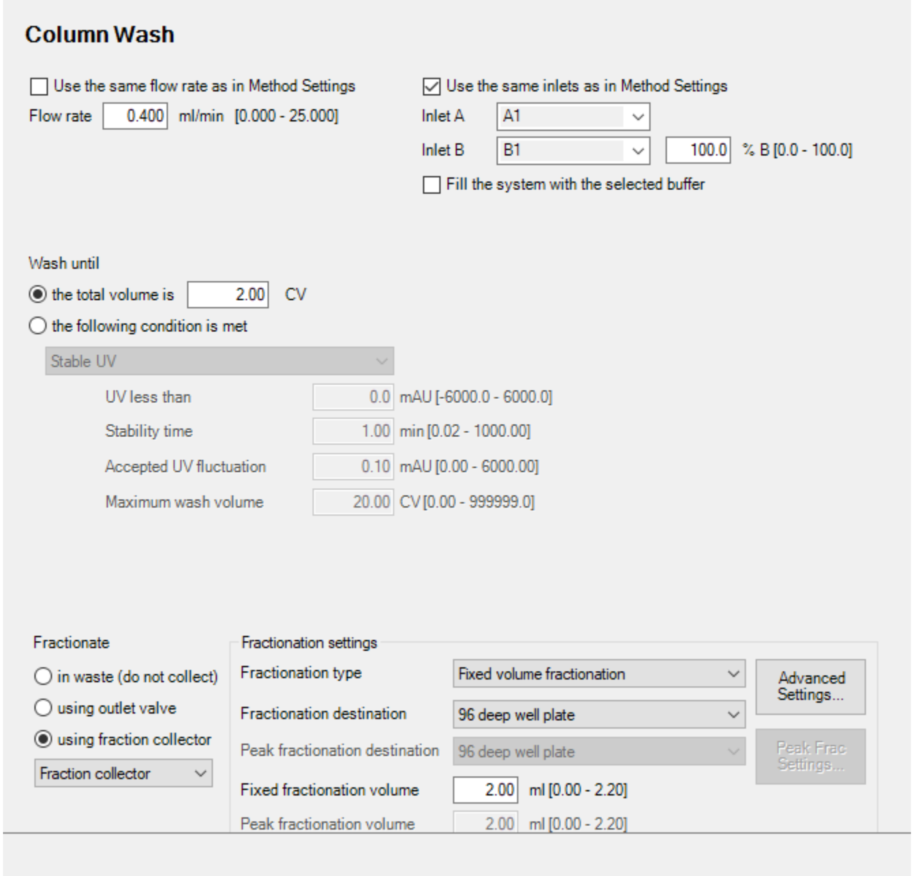

Figure S7: Column wash step with 100% NaCl (2M) solution to remove all leftovers from the column.

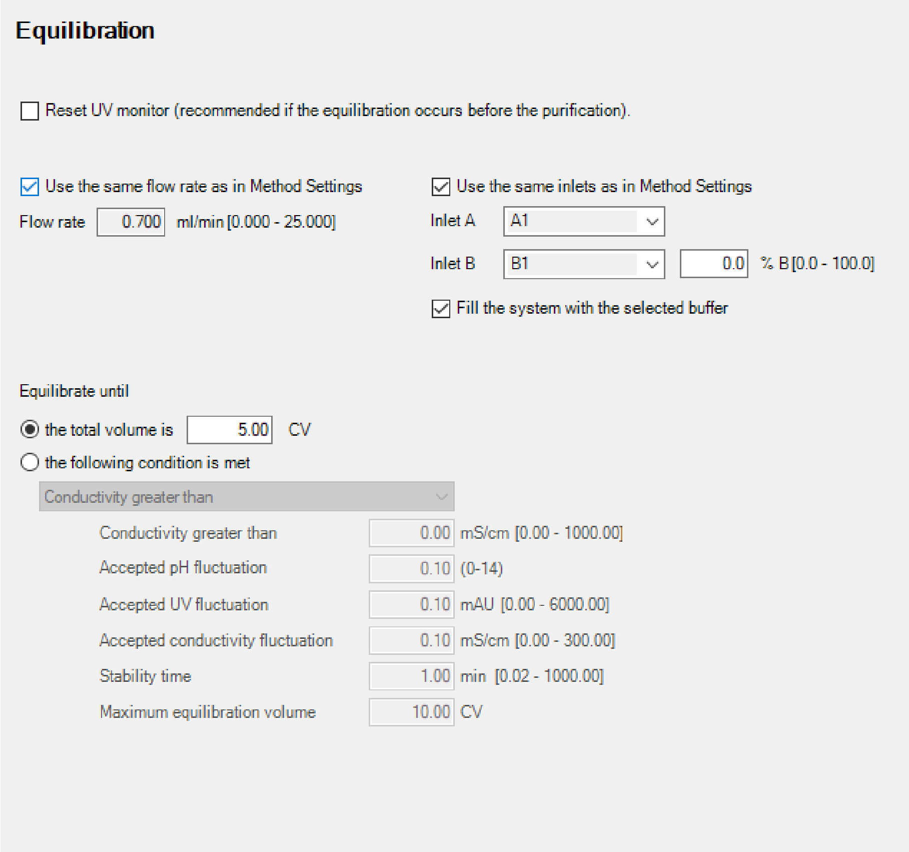

Figure S8: Equilibration step to remove NaCl leftovers and set the milieu to water again.

POST-PREPARATION OF THE OBTAINED PURE CONJUGATE SOLUTION FROM ÄKTA

The obtained solutions contain NaCl due to the purification process. To remove the salt, the solutions were diluted with nuclease-free water and spin filtered with Amicon Ultra-6 mL Centrifugal Filters MWCO 10k for 20 min. This process is repeated three times.

Results and Discussion:

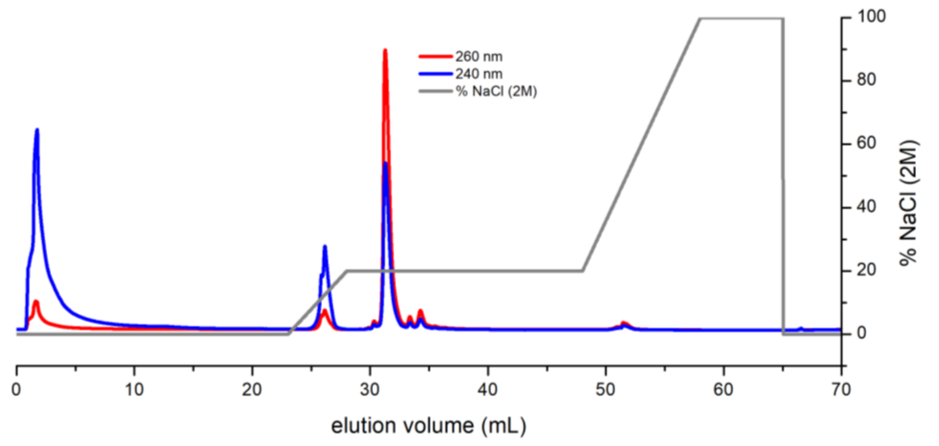

Figure S9: Elution diagram of CP1 reaction solution.

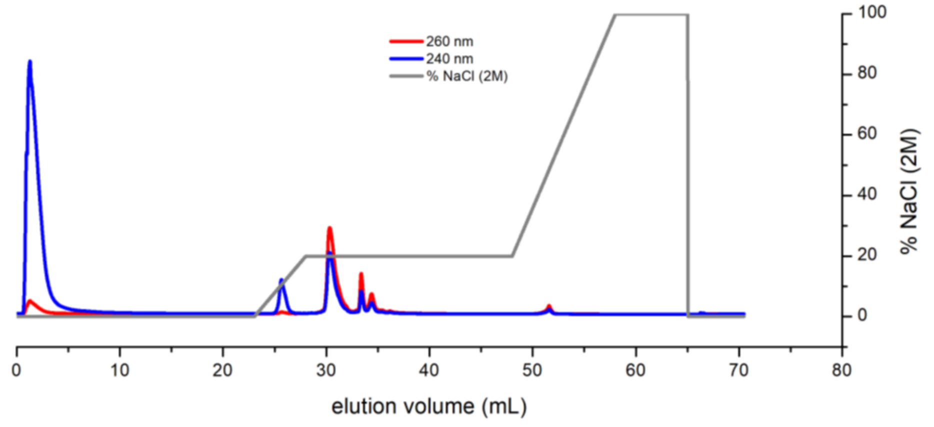

Figure S10: Elution diagram of CP3 reaction solution.

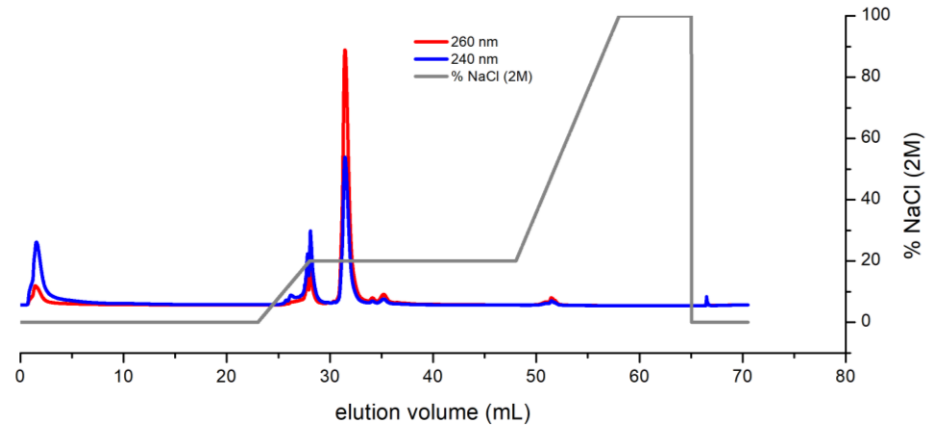

Figure S11: Elution diagram of the CP4 reaction solution.

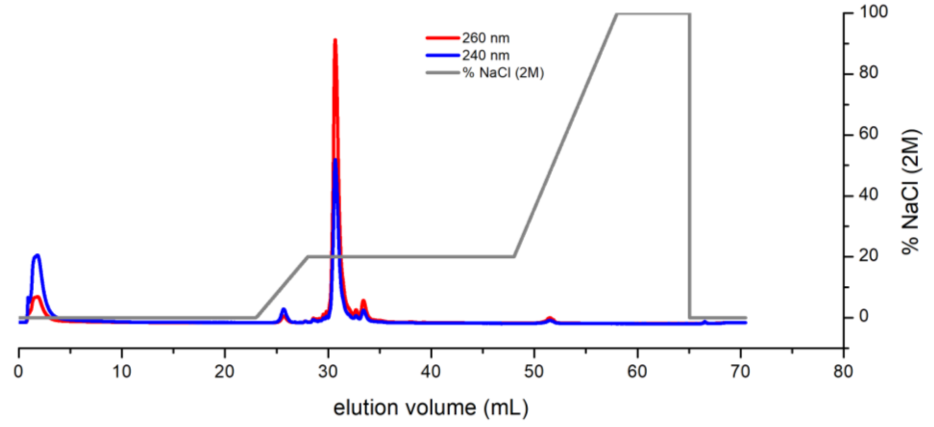

Figure S12: Elution diagram of the CP5 reaction solution.

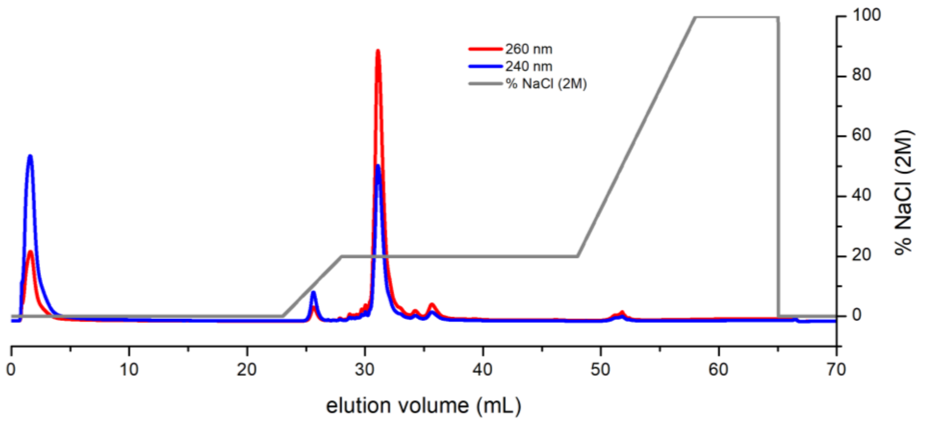

Figure S13: Elution diagram of the CP6 reaction solution.

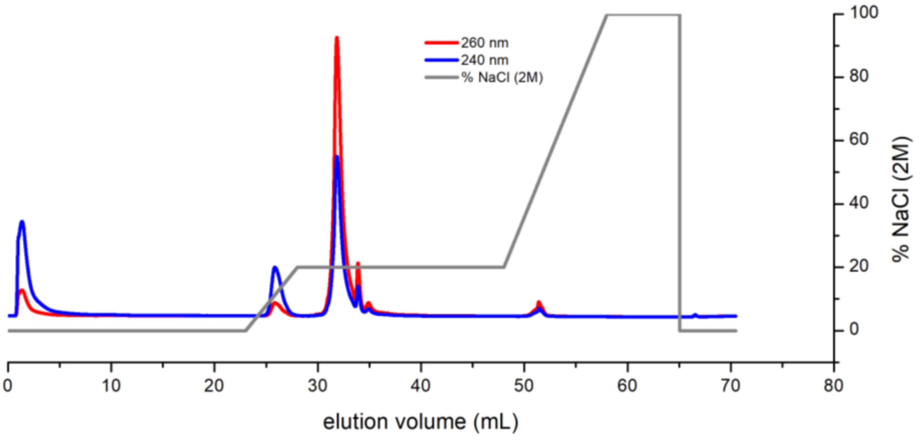

Figure S14: Elution diagram of the CP7 reaction solution.

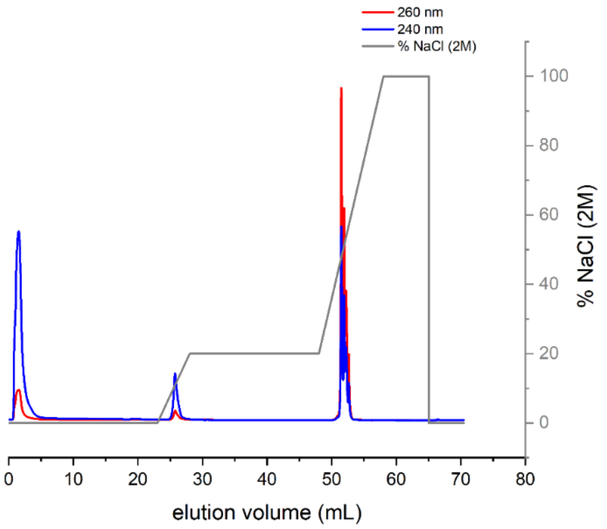

Figure S15: Elution diagram of LCP2 from the first applied gradient. The diagram shows clearly, that the conjugate and also the unreacted oligonucleotide eluate at higher NaCl concentration.

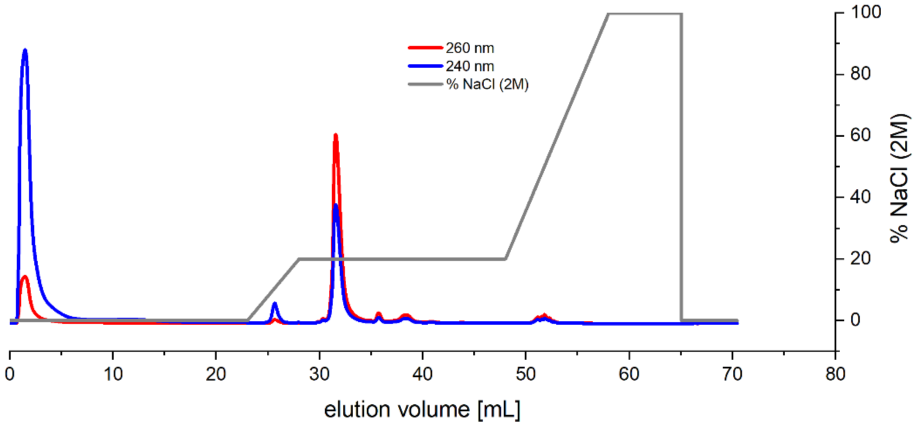

Figure S16: Elution diagram of the CP8 reaction solution.

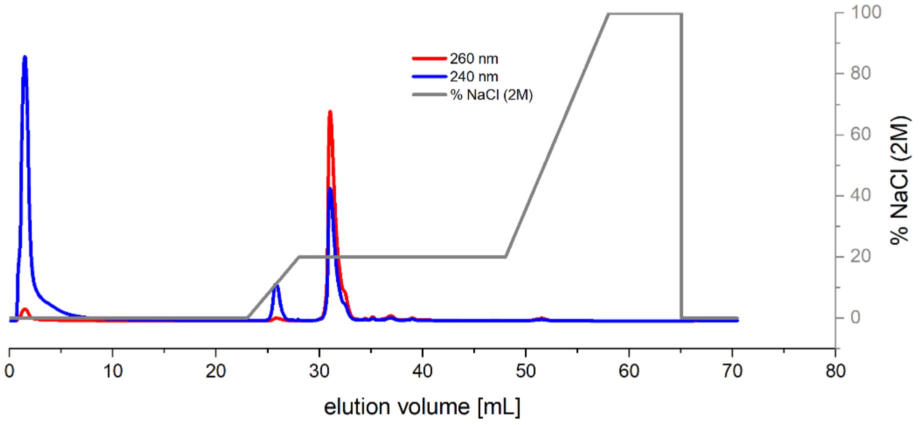

Figure S17: Elution diagram of the CP9 reaction solution.

Table S1: Overview of the synthesized polymers, polymerization parameters and purification solvents.[1]

Polymer	M_w (SEC)	Đ (SEC)	Amount I. Block (SEC)	Used CTA	Polym. solvent	Precipitation solvent	Reaction Temp.	Reaction time
**P(DMA) (P1, P2, P3)**	9649	1.08	/	NHS-DDMAT	dioxane	diethyl ether	70 °C	18 h
	22125	1.08					70 °C	18 h
	48637	1.27					65 °C	4 h
**P(NIPAM) (P4)**	21448	1.12	/	NHS-DDMAT	dioxane	diethyl ether	70 °C	16 h
**P(PEGMA) (P5)**	21090	1.19	/	NHS-CPADB	dioxane	diethyl ether (- 20 °C, phase separation)	70 °C	20.5 h
**P(DAAM-*b*-DMA) (P6)**	26013	1.20	29%	NHS-DDMAT	dioxane	petrol ether	I. Block: 70 °C II. Block: 55 °C	17 h 21 h
**P(HEA) (P7)**	21978	1.28	/	NHS-DDMAT	DMF	diethyl ether	60 °C	17 h
**P(DAAM-*co*-DMA) (P8)**	26614	1.22	2:7 (NMR)	NHS-DDMAT	dioxane	petrolether	70 °C	17 h
**P(NIPAM-*b*-DMA) (P9)**	30445	1.19	43%	NHS-DDMAT	dioxane	diethylether	I. Block: 70 °C II. Block: 65 °C	15 h 17 h

Table S2: Overview of the synthesized polymers, the conversion of the coupling reaction with the 19 base oligonucleotide[1], the yield after purification and the molecular weight/dispersity of the conjugates given by SEC, measured in DMF. Yield after purification is calculated with conversion of the coupling reaction= 100%.

Polymer	M_w (SEC)	Đ (SEC)	Conversion (%)[1]	Yield after purification (%)	M_w conjugate (SEC)	Đ conjugate (SEC)
**P(DMA) (P1, P2, P3)**	9649	1.08	~93	Quantitative	11081	1.21
	22125	1.08	~88	~96	22761	1.21
	48637	1.27	~80	~67	46077	1.34
**P(NIPAM) (P4)**	21448	1.12	~93	~98	23936	1.19
**P(OEGMA) (P5)**	21090	1.19	~94	~63	23600	1.26
**P(DAAM-*b*-DMA) (P6)**	26013	1.20	~96	~60	33841	1.11
**P(HEA) (P7)**	21978	1.28	~94	~70	23278	1.33
**P(DAAM-*co*-DMA) (P8)**	26614	1.22	~69	~93	27791	1.25
**P(NIPAM-*b*-DMA) (P9)**	30445	1.19	~97	~72	31817	1.34

Table S3: Conversion determination using ImageJ intensity calculator to calculate conversion of the CP8 conjugation reaction. Used PAGE gels are shown in Figure S22

	**Intensity**	**Conversion**
**DNA**	307.37	
**CP8**	96.07	69%

Table S4: Conversion determination using ImageJ intensity calculator to calculate conversion and the yield after ÄKTA of the LDNA, LCP2, SDNA and SCP2. Used PAGE gel is shown in Figure 4a

	**Intensity**	**Conversion**	**Yield after ÄKTA if conv. Is 100%**
**LDNA**	2457		
**LCP2**	1137	54%	81.4%
**SDNA**	/		
**SCP2**	/	Quant.	73.5%

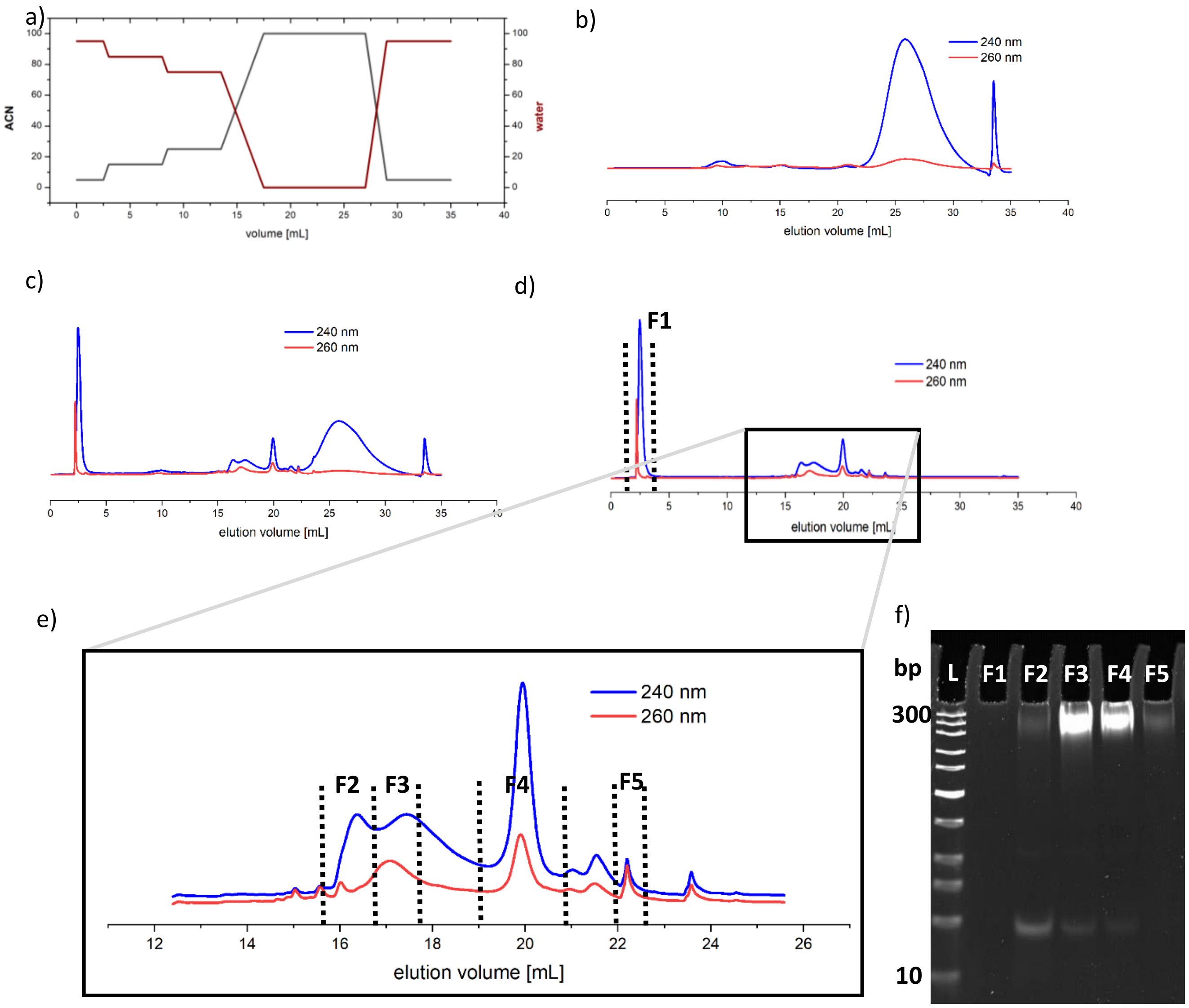

Figure S18: HPLC purification method for the reaction solution **CP2** after coupling. a) Applied stepwise gradient of the purification method with Acetonitrile (ACN) and water as solvents. b) elution diagram with absorption detection of the solvents as baseline. c) elution diagram of the reaction solution purification d) elution diagram with deducted solvent absorption. e) Magnification of the elution diagram in d) for better peak observation. f) PAGE gel (15%) of the respective fractions. Stained with SYBR Gold (2x).

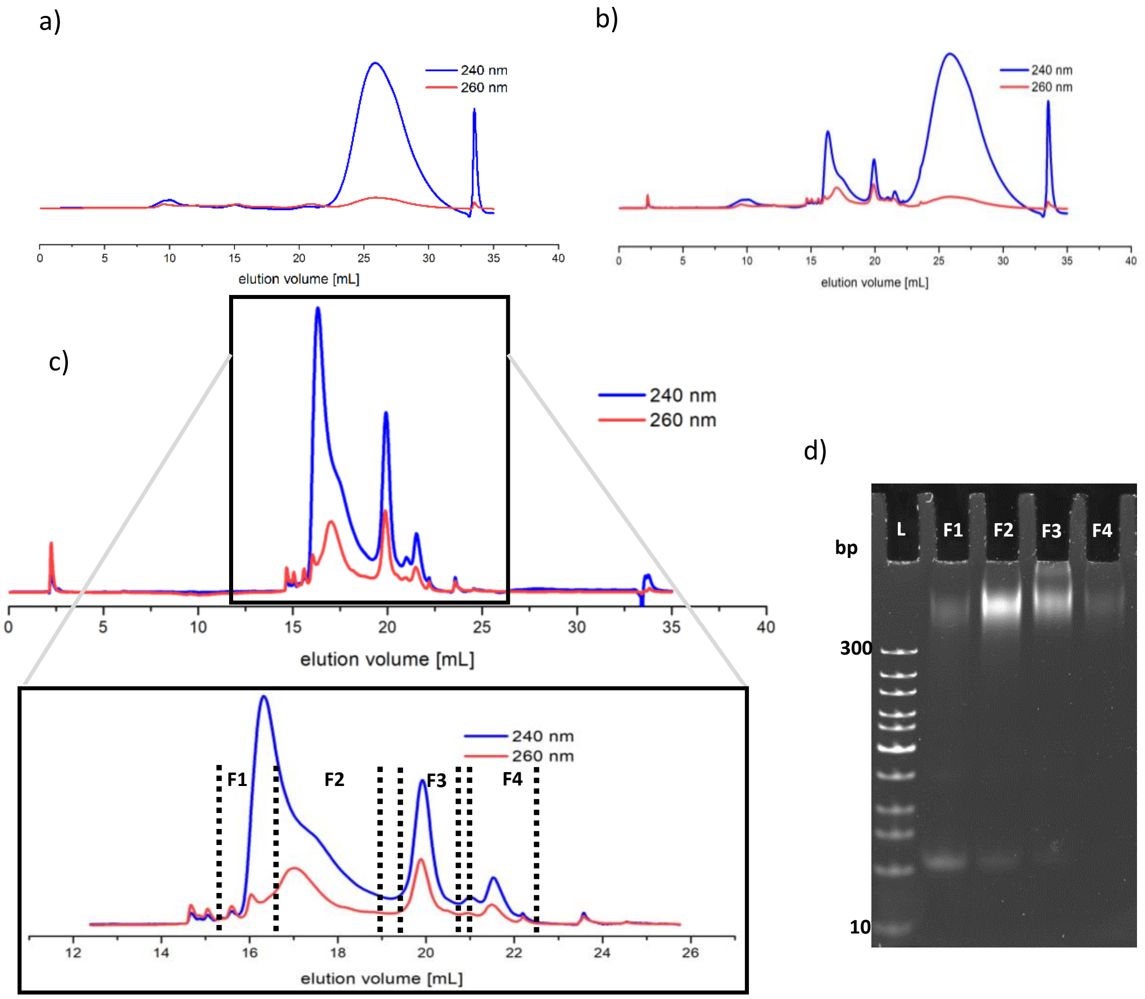

Figure S19: HPLC purification method for the spin filtered reaction solution **CP2** (DMF removed) after coupling with the same stepwise gradient. a) elution diagram with absorption detection of the solvents as baseline. b) elution diagram of the reaction solution purification c) HPLC elution diagram with deducted solvent absorption of the **CP2** reaction solution purification without DMF. Magnification of the elution diagram for the respective fractions. d) PAGE gel (15%) of the respective fractions of the conducted **CP2** reaction solution purification containing DMF. Stained with SYBR Gold (2x).

Table S5: Intensity of the obtained conjugate CP2 from the respective fraction from the PAGE gels shown in Figure S18 and S19 and the calculated proportion from the whole conjugate intensity.

**With DMF:**		
Fraction	Intensity (ImageJ)	Proportion
1	0	0
2	5.955	13.2%
3	17.352	38.4%
4	15.850	35.1%
5	5.980	13.2%
**Without DMF:**		
1	5.362	14.6%
2	14.850	40.4%
3	11.683	31.8%
4	4.846	13.2%

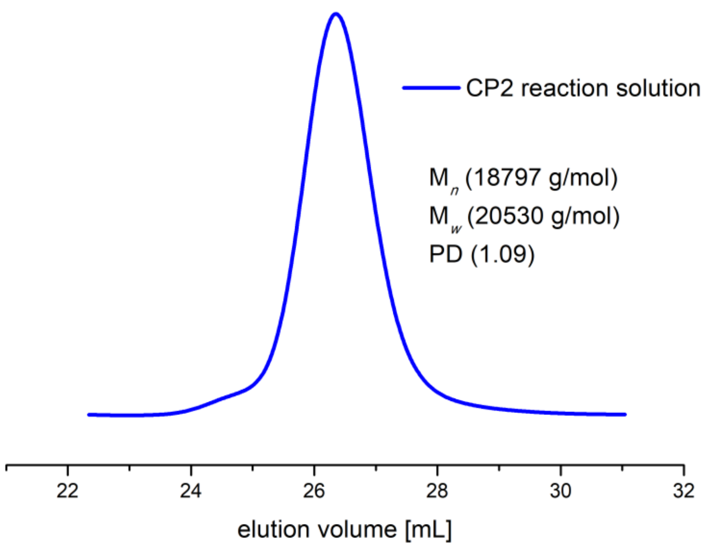

Figure S20: Elution diagrams of the conjugates **CP2** reaction solution measured by DMF SEC using polymethylmethacrylate (PMMA) as standard.

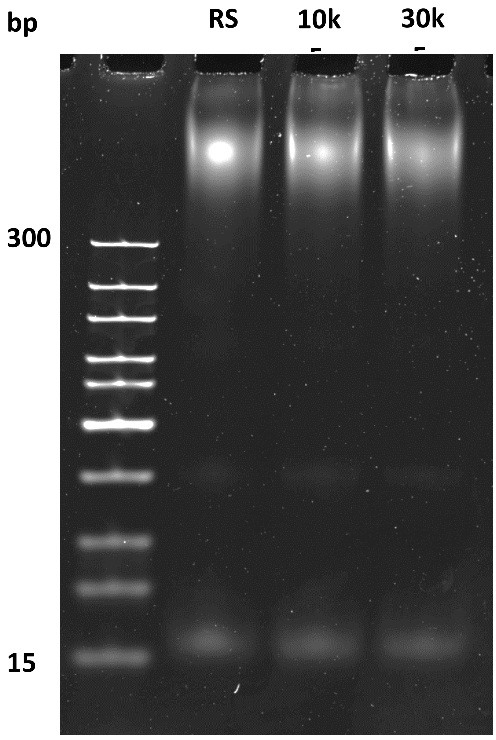

Figure S21: PAGE gel (15%) of the spin filtration of CP2 reaction solution using 10k and 30k cutoff spinfilter. Staine with SYBR Gold (2x).

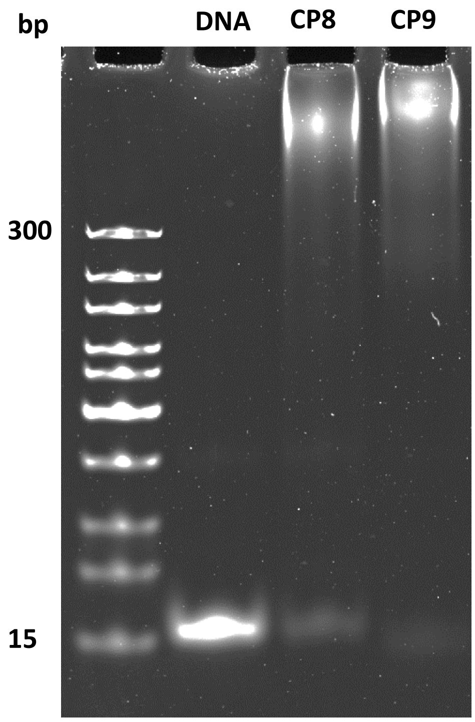

Figure S22: PAGE gel (15%) of the CP8 and CP9 reaction solution. Staine with SYBR Gold (2x).

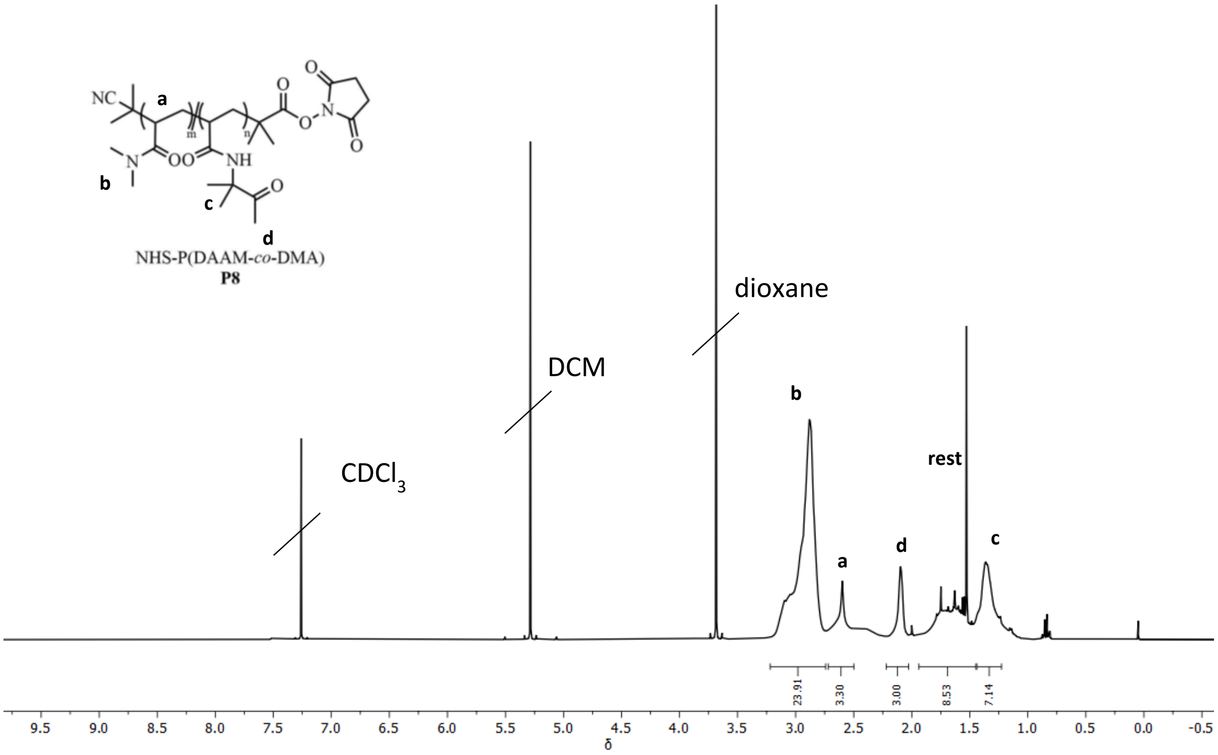

Figure S23: ^{1}H-NMR of P8 synthesized by RAFT polymerization. CTA group was removed with an excess of AIBN.

References

(1) Alleva, N.; Winterwerber, P.; Whitfield, C. J.; Ng, D. Y. W.; Weil, T. Nanoscale patterning of polymers on DNA origami. *J. Mater. Chem. B* **2022**, *10*, 7512–7517.
